# Two Separate Tyrosine-Based YXXL/Φ Motifs within the Glycoprotein E Cytoplasmic Tail of Bovine Herpesvirus 1 Contribute in Virus Anterograde Neuronal Transport

**DOI:** 10.3390/v12091025

**Published:** 2020-09-14

**Authors:** Hocine Yezid, Christian T. Lay, Katrin Pannhorst, Shafiqul I. Chowdhury

**Affiliations:** Department of Pathobiological Sciences, School of Veterinary Medicine, Louisiana State University, Baton Rouge, LA 70803, USA; hyezid08@gmail.com (H.Y.); clay0687@gmail.com (C.T.L.); katrin.pannhorst@fli.de (K.P.)

**Keywords:** BHV-1, glycoprotein E, anterograde neuronal traffic, gE CT domain, YXXL/Φ sorting motifs, compartmentalized primary neurons, microfluidic chambers, reactivation from latency

## Abstract

Bovine herpesvirus 1 (BHV-1) causes respiratory infection and abortion in cattle. Following a primary infection, BHV-1 establishes lifelong latency in the trigeminal ganglia (TG). Periodic reactivation of the latent virus in TG neurons results in anterograde virus transport to nerve endings in the nasal mucosa and nasal virus shedding. The BHV-1 glycoprotein E cytoplasmic tail (gE-CT) is necessary for virus cell-to-cell spread in epithelial cells and neuronal anterograde transport. Recently, we identified two tyrosine residues, Y467 and Y563, within the tyrosine-based motifs _467_YTSL_470_ and _563_YTVV_566_, which, together, account for the gE CT-mediated efficient cell-to-cell spread of BHV-1 in epithelial cells. Here, we determined that in primary neuron cultures in vitro, the individual alanine exchange Y467A or Y563A mutants had significantly diminished anterograde axonal spread. Remarkably, the double-alanine-exchanged Y467A/Y563A mutant virus was not transported anterogradely. Following intranasal infection of rabbits, both wild-type (wt) and the Y467A/Y563A mutant viruses established latency in the TG. Upon dexamethasone-induced reactivation, both wt and the mutant viruses reactivated and replicated equally efficiently in the TG. However, upon reactivation, only the wt, not the mutant, was isolated from nasal swabs. Therefore, the gE-CT tyrosine residues Y467 and Y563 together are required for gE CT-mediated anterograde neuronal transport.

## 1. Introduction

Bovine herpesvirus type 1 (BHV-1) is an alphaherpesvirus that causes abortion, respiratory, and genital infections in cattle [1,2]. Following primary infection of respiratory epithelium, BHV-1 replicates in the nasal epithelium. Subsequently, capsids, along with tegument proteins, enter sensory nerve endings of the trigeminal nerve located in the nasopharynx [1,3]. They are transported retrogradely (from nerve termini to neuron cell bodies) to the trigeminal ganglia (TG), where the virus establishes a lifelong latency [1,3]. Periodic reactivation of the latent virus usually results in nasal viral shedding. In this instance, infectious virus particles are transported anterogradely from the cell bodies in the TG to axon termini in the nasopharynx, which is followed by productive virus replication in the nasal epithelium and nasal virus shedding [1].

Pseudorabies virus (PRV) and BHV-1 envelope proteins, gE, and Us9 homologs are critical for virus anterograde neuronal transport in vitro in primary neurons, and their respective laboratory and natural hosts in vivo [4,5,6,7,8,9,10]. In PRV, targeting of viral structural proteins to axons in vitro required gE, and the finding served as a compelling explanation for the defective anterograde spread of a gE-null mutant in a rat model in vivo [10]. Earlier, we reported that BHV-1 gE-deleted (Δ) and gE cytoplasmic tail (CT)-null viruses, lacking gE carboxy-terminal 125 amino acids (aa), were transported retrogradely, like the wild-type (wt), both in vitro in primary neuron cultures and rabbits and in vivo in cattle [4,11,12]. However, unlike the wt, the gEΔ and gE CT-null mutants had similar anterograde transport defects in vitro (primary neurons) and both in rabbits and calves in vivo [4,11,12]. Also, like the gE-deleted virus, the gE CT-null virus was attenuated in calves. Therefore, the gE CT is one of the functional determinants of virulence and anterograde neuronal transport in cattle, the natural host of BHV-1 [4,12,13].

**Figure 1:**
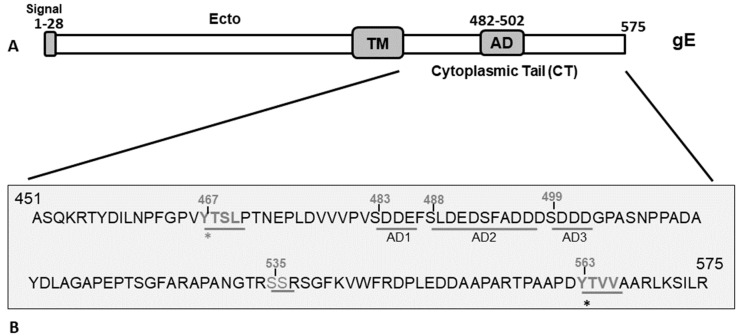
(**A**) Schematic diagram showing the BHV-1 gE protein and its subdomains. The gE extracellular (Ecto), transmembrane (TM), cytoplasmic tail, and acidic (AD) domains are marked. (**B**) Predicted amino acid sequence spanning the gE CT region of BHV-1 (residue 451–575) showing the two tyrosine (Y)-based YXXL/YXX*Φ* motifs (_467_YTSL_470_ and _563_YTVV_566_), serine (S) residues, and an acidic residue cluster (aa 483–502) shown in three sequential segments, AD1, AD2, and AD3, that correspond to the deletion mutants in Figure 2. The gE and its surrounding genes of various gE CT mutants used in this study to elucidate the functional role of gE CT motifs in BHV-1 anterograde neuronal transport were analyzed by sequencing. Further, the mutants were characterized with respect to cell-to-cell spread property, gE subcellular localization, and plaque sizes in non-neuronal cells [17].

Initially, at an early phase (approximately 5–6 h), following infection of polarized epithelial cells by BHV-1, PRV, and herpes simplex virus type 1(HSV-1), the newly synthesized gE homologs are localized in the apical cell membrane. Subsequently, by 8–10 h post-infection (hpi), the gE proteins are internalized from the apical membrane by endocytosis and recycled back to the trans-Golgi network (TGN) [14,15,16,17]). Further, at 8–10 hpi, BHV-1 and HSV-1 gE homologs are redistributed from the TGN to the basolateral cell surface, which is essential for the efficient cell-to-cell spread of the viruses [14,17]. Recently, we mapped the BHV-1 gE CT functional motifs necessary for the gE endocytosis from the apical cell membrane, gE TGN recycling, gE virion incorporation, and gE basolateral cell surface redistribution. Specifically, we determined that the tyrosine-based _467_YTSL_470_ motif (YXXL; Y-tyrosine, X-any amino acid, L-leucine), and, in particular, the tyrosine residue, Y467 (Figure 1), was required for the gE endocytosis and TGN recycling. Whereas, the _563_YTVV_566_ motif (YXXΦ; Y-tyrosine, X-any amino acid Φ-hydrophobic bulky amino acid) and the tyrosine residue Y563 (Figure 1) were essential for gE basolateral cell-surface redistribution (Figure 9 in [17]). Notably, the single tyrosine-based motif _467_YTSL_470_Δ, or only the corresponding Y467A alanine-exchange mutant, and the _563_YTV_565_Δ or Y563A mutant produced similar smaller plaque phenotypes than the wt (Figure 4A in [17]). Whereas, the double _467_YTSL_470_Δ/_563_YTV_565_Δ or Y467A/Y563A mutant produced even smaller plaques than that of their single parental mutant, and the plaques resembled the gE CT-null plaques in size (Figure 4A in [17]). Based on these findings, we concluded that the gE-CT tyrosine-based motifs _467_YTSL_470_ and _563_YTVV_566_, particularly the residues Y467 and Y563 together, contributed to BHV-1 cell-to-cell spread in epithelial cells [17].

As noted above, the anterograde neuronal transport function of gE mapped within the 125 aa of the gE CT [12]. However, specific motifs within the gE CT subdomains were not functionally characterized for the anterograde neuronal spread. As noted above, the two tyrosine-based motifs and the corresponding tyrosine residues, Y467 and Y563 mutations within the gE CT were linked functionally to the gE subcellular localization and BHV-1 cell-to-cell spread defects in MDBK cells in vitro [17]. Therefore, we hypothesized that these two tyrosine-based motifs might also display essential functional role in the anterograde neuronal transport of BHV-1.

To test our hypothesis, we first analyzed anterograde neuronal transport properties of various gE CT subdomain mutant viruses in vitro in a compartmentalized primary neuron culture system. The analyses revealed that, while the gE CT alanine-exchange mutants, Y467A, or Y563A mutants displayed significantly diminished anterograde axonal spread, the double-alanine-exchanged Y467A/Y563A mutant virus displayed a complete loss of the anterograde neuronal transport property. Further, characterization of the double Y467A/Y563A mutant virus in rabbits in vivo revealed that the double mutant virus replicated efficiently in the nasal epithelium of rabbits during primary infection transported retrogradely to the TG neurons and established latency. However, following reactivation of the latent virus in the TG neurons, the mutant virus did not transport anterogradely to the nasal epithelium, and there was no nasal virus shedding.

## 2. Materials and Methods

### 2.1. Cells

The Madin-Darby bovine kidney (MDBK) cell line was maintained in Dulbecco’s modified Eagle’s medium (DMEM) supplemented with 5–10% heat-inactivated fetal bovine serum (FBS).

### 2.2. Viruses

The construction of full-length infectious BAC clone of BHV-1 wt BAC and characterization of the reconstituted BAC containing (*) and BAC-excised BHV-1 wt* and BHV-1 lox viruses, respectively, have been described elsewhere [12]. The predicted amino acid sequence of BHV-1 gE CT is shown in Figure 1. BHV-1 gE CT mutant viruses, gE CT-null, AD1Δ, AD2Δ, AD3Δ, _535_SS_536_Δ, _457_YDIL_460_Δ, Y467A, Y563A, and double-alanine-exchanged Y467A/Y563A, were generated by en passant two-step mutagenesis of the BHV-1 wt BAC infectious clone [12,17]. Low-passage viral stocks were maintained at −80 °C.

### 2.3. Primary Neuronal Cultures and Virus Infection in the Microfluidic Chamber System

A microfluidic chamber system with a microgroove length of 450 µm and a width of 10 µm (Xona Microfluidics LLC, CA, USA) was used. Detailed methods of microfluidic chamber assembly, primary rabbit dorsal root ganglionic (DRG) neuronal cultures, and viral infections for the neuronal transport study were described elsewhere [11]. Briefly, 48 h before virus infection when the DRG cultures were 19 to 20 days old, MDBK cells were seeded in the axonal compartment. First, freshly prepared neuron culture media (neuron media) was added in two wells (170 µL/well) of the neurite chamber. Then, the two wells of the soma chamber containing neuronal cell bodies were infected with 120 µL of each virus suspension in neuron-media, containing approx. 1.0 × 10^7^ plaque-forming units (PFU), at a multiplicity of infection (MOI) of approximately 50. After virus adsorption (usually after 90 min), the media in the soma compartment was replaced with 140 µL of fresh neuron culture medium. The volume difference between the soma and axonal chambers maintained a hydrostatic pressure gradient, which prevented the diffusion of infectious virions from the soma chamber through the microgroove into the axonal chamber. The microfluidic chambers were then incubated further in a CO_2_ incubator until fluorescent microscopy or the harvesting of infected neurons or MDBK cells for viral plaque assay. Photographic images of infected neurons (soma chamber) and MDBK cells in the neurite chamber were captured using an Olympus Fluorescent microscope attached to a digital camera system. At 74 hpi, infected neurons with media (soma chamber) and MDBK cells with media (neurite chamber) were harvested separately and stored at −80 °C. Viral plaque assay was performed as previously described [18].

### 2.4. Rabbit Experiments

Animal infection, handling, sample collection, and euthanasia protocols were approved previously by the LSU Institutional Animal Care and Use Committee. Two groups of rabbits (*n* = 8 each) were either infected with BAC excised BHV-1 lox wt [12] or the gE Y467A/Y563A mutant virus [17]. Rabbits were first sedated by intramuscular (IM) injection of Rompun (Bayer Corp; 5 mg/kg), and then infected with 2 × 10^7^ PFUs by intranasal instillation (1 × 10^7^ PFUs/nostril). At 28 days post-infection (dpi), rabbits in each group were injected with dexamethasone (2.8 mg/kg by I/M) for five days to reactivate the latent viruses [12]. During the acute infection (1–14 dpi) and latency (15–28 dpi), nasal swabs were collected on 0, 3, 5, 7, 9, 15, 21, and 28 dpi. Following dexamethasone (dex)-induced reactivation on 28 dpi (dex 0), nasal swabs were collected on days dex 0, dex 4, dex 5, and dex 7. Nasal swabs were stored at −80 °C. At seven days post-reactivation (dex 7), rabbits were euthanized and TGs were collected for RNA isolation. Briefly, each TG was dissected into three segments, snap-frozen in liquid nitrogen, and stored at −80 °C. Virus isolation from the nasal swabs and virus titration by plaque assay were performed as described earlier [5,12].

### 2.5. RNA Extraction and Reverse-Transcriptase PCR (RT-PCR)

Briefly, RT-PCR experiments to detect BHV-1 major capsid protein (virion protein 5 or VP5) and cellular glyceraldehyde-3-phosphate dehydrogenase (GAPDH) transcriptions were performed using a one-step RT-PCR kit (Qiagen Inc.—MD, USA) as described earlier with slight modifications [12]. Briefly, total RNAs were extracted from TG of euthanized rabbits using RNA easy extraction kit (Qiagen), followed by extensive digestion with RNase-free DNase I. CDNA was generated from 1 µg total RNA using cDNA high-capacity reverse transcription kit (Quigen one-step RT-PCR kit) following the manufacturer’s protocol. BHV-1 VP5-specific 251 bp fragment was amplified by PCR from the cDNA using the forward and reverse primer pairs 5′-tgcggtctgcgagttcatc-3′ and 5′-cgccgctcatgttgtactg-3′, respectively. As an internal control, GAPDH sequence (841 bp) was amplified using the forward and reverse primer pairs 5′-tgttccagtatgattccaccc-3′ and 5′-tccaccaccctgttgctgta-3′, respectively. As positive and negative controls for VP5, BHV-1-infected, and mock-infected MDBK cellular RNA were used for RT-PCR reaction as above. As a control for DNA contamination in RNA samples, a PCR reaction lacking the RT step was performed using the BHV-1 VP5-specific primers. The gel image of VP5- and GAPDH-specific PCR products were captured by agarose gel electrophoresis. The amplified VP5 band was quantified by densitometric analysis and normalized to the corresponding GAPDH band for each sample using the Quantity one software (Bio-Rad Laboratories, CA, USA).

### 2.6. Statistical Analysis 

Statistical significance of nasal virus shedding titers and VP 5 transcription levels in the TGs of rabbits were calculated using two-sided Student’s *t*-tests using Graph Pad Prism 8. The *p*-values in Figure 3 and Figure 4E are indicated as * *p* < 0.05, ** *p* < 0.01, and nonsignificant (ns) for *p* > 0.05.

## 3. Results

### 3.1. Both gE-Y467A and gE-Y563A Mutants Showed Diminished Anterograde Axonal Transport in Primary Neuron Culture, While the Double Y467A/Y563A Mutant Virus, Like the gE CT-Null Virus, Was Unable to Transport Anterogradely

The BAC-excised gE CT-null, and gE CT subdomain mutant viruses AD1Δ, AD2Δ, AD3Δ, _535_SS_536_Δ, _457_YDIL_460_Δ, _467_YTSL_470_ Δ, Y467A, _563_YTV_565_Δ, YTV rescued, Y563A, and double-alanine-exchanged Y467A/Y563A were characterized previously by sequence analysis by their phenotypes in MDBK cells, especially the growth properties, plaque sizes, and cell-to-cell spread characteristics [12,17]. The mutants with smaller plaque phenotypes and cell-to-cell spread defects (Y467A or _467_YTSL_470_Δ, Y563A or _563_YTV_565_Δ, and double-alanine-exchanged Y467A/Y563A) were analyzed further for gE cell-surface expression, gE endocytosis and TGN recycling, gE basolateral cell-surface localization, gE envelope incorporation, and gE-VP22 tegument protein interaction [17]. Based on the results reported in detail recently, we concluded that the demonstrable phenotypic and functional effects of _467_YTSL_470_Δ or Y467A, _563_YTV_565_Δ, or Y563A and Y467A/Y563A mutations were gE sequence-specific and not due to some unintended secondary mutations of the neighboring genes [17].

**Figure 2:**
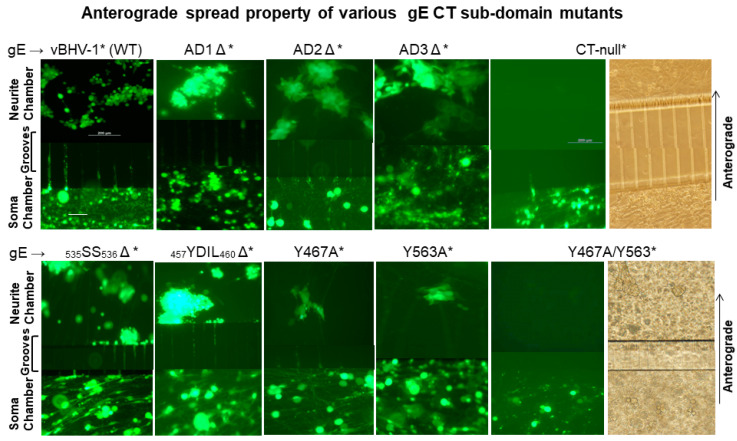
Anterograde spread of BHV-1 wt and various gE CT mutant viruses in compartmentalized primary neuronal culture. DRG neurons seeded in the soma compartment of microfluidic chambers were allowed to grow for two weeks, by which time the axons extended across the grooves to the neurite chamber and formed an extensive network there. MDBK cells were plated in the neurite chamber two days before the virus infection of the neurons in the soma chamber. Reconstituted BAC containing (*) vBHV-1WT*, gE CT-null*, gE ΔAD1*, gE ΔAD2*, gE ΔAD3*, gE _535_SS_536*_, gEΔ _457_YDIL_460_*, gE Y467A, gE Y563A, and gE Y467A/Y563A viruses at an MOI of 50 (approximately 50 PFUs/neuron) were used for each virus infections. Representative fluorescent microscopic images shown were taken at 74 hpi. (Scale bar: 200 μm).

Previously, we reported that BHV-1 gEΔ and gE CT-null viruses were transported retrogradely, equally efficiently like the wt both in vitro in primary neuron cultures, and in rabbits and cattle in vivo. However, the gEΔ and gE CT-null mutants had anterograde transport defects both in compartmentalized primary neuron cultures in the microfluidic chamber system, as well as in rabbits and calves [4,11,12]. Therefore, we only determined the anterograde neuronal transport of the gE CT subdomain mutants in the microfluidic chambers compared to that of the gE CT-null and wt viruses. The reconstituted, infectious BAC containing (*) BHV-1 wt*, gE CT-null*, gE AD1Δ*, AD2Δ*, AD3Δ*, _535_SS_536_Δ*, _457_YDIL_460_Δ*, Y467A*, Y563A*, and the double Y467A/Y563A* mutant viruses express the diffusible green fluorescent protein (GFP) [11,17]. Therefore, virus infection and replication in neuronal cell bodies (soma chamber), and virus anterograde axonal transport (across the grooves) to the corresponding neurite chamber were visualized by GFP expression. The results were documented by live-cell images of the compartmentalized neuron cell bodies, and their axons were associated with MDBK cells in the neurite chamber (Figure 2).

Analysis of soma chamber for virus infection from 12–74 hpi indicated that all the viruses listed above, including the gE CT* mutants, infected and replicated similarly in the neuronal cell bodies. As depicted in Figure 2, at 74 hpi (immediately before harvesting), the number of infected neurons, as well as the intensity of GFP-expression in the soma chambers inoculated with wt, gE CT-null*, and gE CT*-mutant viruses, were comparable. Also, at 74 hpi, virus yields from the soma chambers were similar regardless of the wt and mutants, which ranged between 3–4 × 10^4^ PFUs/100 µL. The results also showed that as early as 24 h after the soma chamber infection, BHV-1 wt*, and the gE-CT* mutants- AD1Δ*, AD2Δ*, AD3Δ*, _535_SS_536_Δ*, and _457_YDIL_460_Δ* viruses transported anterogradely along the axons in the grooves, to the corresponding neurite chamber and produced multiple GFP expressing plaques or islands of MDBK cells. Notably, even at 72–74 h after the soma chamber infection, only a single GFP expressing plaque was detected in the corresponding neurite chamber of the Y467A* and Y563A* mutants, respectively. Remarkably, no GFP plaque could be visualized in the neurite chambers of double Y467A/Y563A* and gE CT-null* mutant virus at this time (Figure 2). We obtained very similar results with the individual _467_YTSL_470_Δ* and _563_YTV_565_Δ*, and double _467_YTSL_470_/_563_YTV_565_Δ* mutant viruses. From these results, we concluded that Y467A* and Y563A* mutants showed significantly diminished anterograde axonal transport, while, like the gE CT-null*, the Y467A/Y563A* mutant was incapable of anterograde axonal transport.

Attempts to quantify viruses by plaque assay from the neurite chambers of wt, as well as the gE mutant viruses, were unsuccessful. These experiments were repeated twice with very similar results.

### 3.2. In Vivo Anterograde Neuronal Transport Property of Dual Y467A/Y563A Mutant and BHV-1 Lox (wt) Viruses in Rabbits

Previously, we reported that both in calves and rabbits, regardless of wt, gEΔ, or gE CT-null viruses, the viruses replicated similarly in the nasal epithelium for the first 5–6 dpi, and were transported retrogradely via the trigeminal nerve to the TG neurons and established latency. Also, the three viruses reactivated similarly in the TG neurons following the dexamethasone-induced immunosuppression. However, only the wt virus was isolated from the nasal swabs [4,12]. Based on these results of animal experiments and defective neuronal anterograde transport of the gEΔ and gE CT-null viruses in the primary neuron cultures [11], we concluded that gE, and particularly the gE CT residues 451–575, played an essential role in the neuronal transport of BHV-1. As detailed above, like the gE CT-null virus, the double Y467A/Y563A mutant virus did not transport anterogradely from the neuron cell bodies, in the soma chamber to the axon termini in the neurite chamber. Therefore, to validate the in vitro anterograde neuronal transport defect of the Y467A/Y563A mutant virus in vivo, we tested its nasal virus shedding in rabbits compared to that of parental BAC-excised wt virus (BHV-1 lox) following the primary infection, during latency, and after reactivation. In addition, we investigated VP5 transcription in the TG neurons of Y467A/Y563A mutant- and BHV-1 lox-infected rabbits following the latency reactivation. VP5 is coded by the late gene (UL19). Transcription of alphaherpesvirus late genes, in the TG neurons, occurs following early protein expression, which are proteins involved in DNA replication. Therefore, the transcription of VP5 in the TG is dependent on virus DNA replication [19,20,21]. In these cases, the detection of VP5-specific transcription in the TG neurons of rabbits infected with either wt or Y467A/Y563A mutant is an indirect confirmation that the latent Y467A/Y563A DNA replicated in the TG following the latency reactivation.

**Figure 3:**
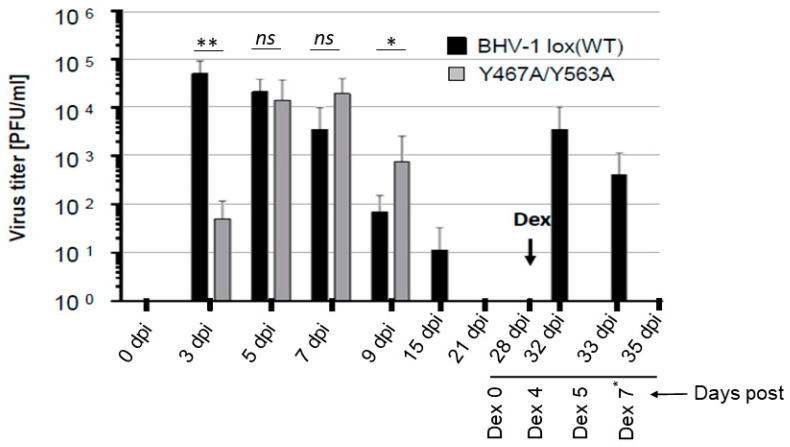
Nasal virus shedding of BHV-1 lox (wt) or BHV-1 Y467A/Y563A in infected rabbits. Quantification of BHV-1 wt and BHV-1 Y467A/Y563A viruses present in nasal swabs of rabbits during the primary infection, latency, and dexamethasone-induced latency reactivation. The viruses were isolated at the indicated time points and titrated by plaque assay on MDBK cells. The data represent averages and standard deviations for each group. Dex refers to the onset of dexamethasone injection. The *p*-values are indicated as * *p* < 0.05, ** *p* < 0.01. and nonsignificant (*ns*) for *p* > 0.05. Note that on Dex 7* day, nasal swabs were collected only from the rabbits infected with Y467A/Y563A double mutant virus.

The results showed that at 3 dpi, virus titer in the nasal swabs of Y467A/Y563A-infected rabbits was 30-fold less to that of the wt. However, at 5 dpi, virus titers in the nasal swabs of rabbits either with wt or Y467A/Y563A mutant virus were very similar, and both at 7 dpi and 9 dpi, the gE mutant’s nasal virus titers were slightly higher than the wt. Nevertheless, the nasal virus shedding in the wt virus-infected rabbits lasted a few days longer than the gE mutant virus (until 15 dpi). As expected, during latency, at 21 dpi and 28 dpi, the virus was not isolated from the nasal swabs for both wt and mutant virus-infected rabbits. Following dexamethasone (Dex) injections, both at four and five days after the first injection, the wt virus was readily isolated from nasal swabs of all the infected rabbits, with titers ranging approximately between 10^3^–10^4^ PFU/mL (Figure 3). However, the Y467A/Y563A mutant virus-infected rabbits remained negative until seven days-post-DEX when the rabbits were euthanized (Figure 3).

**Figure 4:**
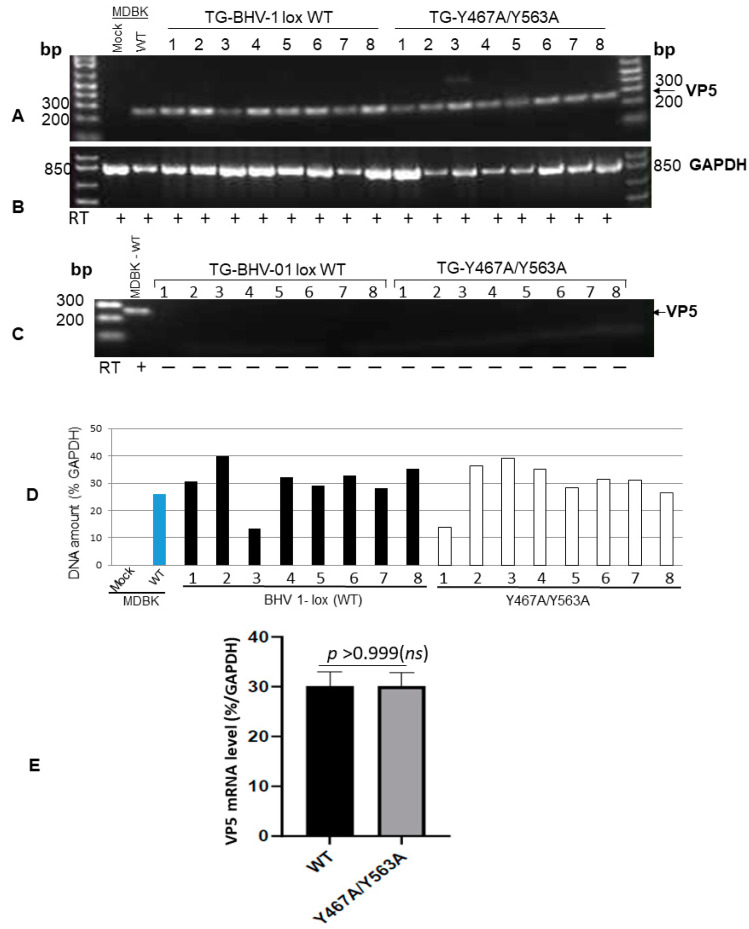
RT-PCR amplification of BHV-1 VP5 (251-bp)-specific mRNA sequence from TGs of rabbits following reactivation. (**A**) Amplification of 251 bp VP5 transcript sequence from the TGs of BHV-1 lox wt- and gE Y467A/Y563A-infected rabbits following the latency reactivation. The positive control is the VP5 transcript amplified from wt virus-infected MDBK cells, and the negative control is uninfected MDBK cellular RNA. (**B**) As an internal control, the GAPDH (841-bp) transcript sequence was amplified using the primer pairs 5′-tgttccagtatgattccaccc-3′ (forward) and 5′-tccaccaccctgttgctgta-3′ (reverse) from each respective TG RNA sample. (**C**) Verification of the virus genomic DNA contamination: The RT-PCR reaction was carried out as in (**A**) but without the reverse transcription step using the VP5 specific primer pairs 5′-tgcggtctgcgagttcatc-3′ (forward) and 5′-cgccgctcatgttgtactg-3′ (reverse). (**D**) VP5-specific mRNA levels in TG of wt and mutant-infected rabbits were normalized to the intracellular GAPDH gene transcription levels using the Quantity One software (Bio-Rad laboratories). (**E**) Statistical analysis of VP-5 transcription in TGs of rabbits infected with BHV1 wt versus gE Y467A/Y563A mutant following latency-reactivation. Data represent the mean ± SEM. The *p* values, *p* > 0.05 = nonsignificant (*ns*).

To confirm that both the wt and Y467A/Y563A mutant virus reactivated in the TGs of latently infected rabbits following dexamethasone injections, we determined the VP5 gene-specific mRNA transcription levels in the TGs following euthanasia. The results depicted in Figure 4 validate that regardless of wt or Y467A/Y563A mutant virus, the level of VP5-specific transcription in the TGs of rabbits was similar. Therefore, both wt and the Y467A/Y563A mutant viruses reactivated from latency and replicated in the TG with the same efficiency. However, the anterograde transportability of the Y467A/563A virus from the TG neurons to nerve endings in the nasal mucosa was abrogated.

In conclusion, both BHV-1 wt and the Y467A/Y563A mutant viruses were transported retrogradely from nerve endings in the nose to cell bodies in the TG and established latency. Upon reactivation, both viruses replicated in the TG equally efficiently. However, due to the loss of anterograde transportability, as demonstrated visibly in the primary neurons (Figure 2), the Y467A/563A mutant virus was not transported to and shed from the nasal mucosa. Therefore, the two tyrosine-based _467_YTSL_470_ and _563_YTVV_566_ motifs within the gE CT of BHV-1 are required for, and together contribute, to efficient anterograde neuronal transport of the virus.

## 4. Discussion

Earlier, we reported that BHV-1 gE-deleted and gE cytoplasmic tail-truncated mutant viruses established latent infection in the TG of calves following intranasal infection. Additionally, we reported that in both cases, the virus replicated in the TG following dexamethasone (Dex)-induced reactivation of the latent virus but was not isolated from nasal and ocular swabs [4,11,12]. From these indirect results, we inferred that carboxy-terminal gE amino acid residues 451–575, which are also defined as the gE CT residues, were not required for retrograde axonal transport of the virus from the sensory nerve ending in the nose to neuronal cell bodies in TG. However, it was needed for the anterograde axonal transport of the virus from neuronal cell bodies in the TG to their nerve endings in the nose. Subsequently, we validated our results above by demonstrating, visually, in compartmentalized primary neuron cultures in vitro, that regardless of BHV-1 wt, BHV-1 gEΔ, and gE CT-null viruses, all three viruses transported retrogradely equally efficiently. However, only the wt virus was transported anterogradely from the neuron cell bodies to the axon termini in the compartmentalized neuronal culture system [11].

Recently, we mapped the BHV-1 gE CT functional domains essential for BHV-1 cell-to-cell spread function in MDBK cells in vitro. Specifically, we determined that the two tyrosine-based motifs within the gE CT, _467_YTSL_470_ and _563_YTVV_566_, or their corresponding residues Y467 and Y563, collectively contributed to the gE-mediated efficient cell-to cell-spread function. Specifically, the individual Y467A and Y563A mutants produced smaller plaques relative to the gEΔ virus but slightly larger than the gE CT-null virus plaques. However, when both the mutations were incorporated in a single virus, the Y467A/Y563A mutant produced gEΔ or gE CT-null virus-size plaques [17]. Further, we showed that these two motifs differ functionally with respect to their role in gE recycling from the cell-surface to TGN or gE translocation from the TGN to the basolateral cell surface. Nevertheless, their ultimate and predominant collective effect has been to facilitate efficient virus cell-to-cell spread defects [17].

In this study, our goal was to determine whether the gE CT mutants, especially the two Y467A and Y563A mutations, either individually or in combination (double Y467A/Y563A mutation), play a critical role in the anterograde axonal transport of BHV-1. We achieved this goal by comparing anterograde transport of various gE CT mutants in vitro in compartmentalized primary neuron culture. We determined that two different gE CT mutant viruses, containing either Y467A or Y563A exchanges, had similarly impaired anterograde neuronal transport. Interestingly, when both mutations were incorporated in a single virus, the double Y467A/Y563A mutant virus lost the anterograde neuronal transport property completely. Based on these results, we selected the double Y467A/Y563A mutant virus to test further and validate its loss of anterograde transport property in vivo in rabbits. As expected, the Y467A/Y563A mutant virus, like the wt, replicated in the nasal mucosa of rabbits for 5–7 days, and subsequently transported retrogradely up the axons to the TG neurons efficiently. In the TG, the double mutant virus also established latency, reactivated, and replicated like the wt, as evidenced by the VP5 mRNA transcription levels. However, only the wt virus, not the Y467A/Y563A mutant virus, shed from the nose following reactivation. As noted above, VP5 is a late viral protein and is required for virus capsid assembly [20]. Even though VP5 has a leaky late profile in vitro, in the epithelial cells, VP5 (UL 19) gene transcription in the TG neurons requires beta (early) gene expression and DNA replication [20,21]. Therefore, detection of VP5-specific transcription in the TG neurons of rabbits infected with either wt and gE CT-null viruses has been used as an indirect demonstration of virus reactivation and replication in the TG neurons [12].

The anterograde axonal transport from TG neurons to nerve endings in the nasal mucosa of rabbits and calves cannot be visualized microscopically. Therefore, the anterograde spread defect of the double mutant virus in vivo can only be explained in light of the results obtained from (i) the well-established anterograde neuronal transport assays in compartmentalized primary neuron culture system, (ii) the semi-quantitative RT-PCR assays for the late gene UL 19 (VP5) transcription in the TGs of rabbits following the dexamethasone-induced reactivation of the latent viruses, and (iii) virus shedding in the nose following the latency-reactivation. Using these parameters, we demonstrated that both the latent wt and double mutant viruses reactivated and replicated in the TG very similarly. However, only the wt virus shed in the nose following reactivation. Therefore, the lack of nasal virus shedding in the case of rabbits infected with the double Y467A/563A mutant virus can only be due to the loss of gE- mediated anterograde neuronal transport function [4,12].

As noted above, the double Y467A/Y563A mutant virus had similar cell-to-cell spread defects and produced, similarly, smaller size plaques in the MDBK cells, as was in the cases of gEΔ and gE CT-null viruses [17]. Notably, the tyrosine residue, Y467A exchange mutant virus, had defective gE endocytosis and TGN subcellular localization, while the Y563A mutant virus had defective basolateral cell-surface localization [17]. Based on these results, we concluded that BHV-1 gE subcellular trafficking in non-neuronal cells in vitro is contributed by the two different tyrosine-based motifs _467_YTSL_470_ and _563_YTVV_566_ within the gE CT. Together, these two motifs, and specifically the tyrosine residues Y467 and Y563, promoted the efficient virus cell-to-cell spread. Based on several earlier studies, the wt, gEΔ, gE CT-null, and double gE tyrosine mutant viruses replicated with a similar kinetics and virus yield in MDBK cells [12,13,17]. Therefore, regardless of wt or the gE mutant viruses, their replication in the cell and release from the apical cell membrane were not affected. However, when the apically released viruses were confined by overlaying the infected cell monolayers with a viscus media or media containing agar, the gEΔ, gE CT-null, and double Y467A/Y563A mutant viruses produced very similar smaller-sized plaques compared to that of the wt, because they all had the identical cell-to-cell spread defect [12,13,17]. As previously noted, at early times post-infection, BHV-1 and HSV-1 gE homologs were expressed on the apical cell membrane of epithelial cells. At a later time, they were internalized from the cell membrane, recycled to the TGN, and redistributed from the TGN to the basolateral cell membrane. Notably, the gE-redistribution to the basolateral cell surface was essential for efficient cell-to-cell spread of the viruses [14,17].

The TGN serves as the primary sorting station for packaging apical and basolateral cargo molecules into their respective transport vesicles [22]. The basolateral sorting of many membrane proteins, as well as specific TGN proteins such as TGN38/46 and the furin cycle to the basolateral membrane, is dependent on interactions of critical tyrosine- or leucine-based motifs in the cytoplasmic tail with specific adaptor proteins. These motifs resemble sequences that specify rapid internalization from the cell surface, suggesting a relationship between basolateral sorting and clathrin-mediated endocytosis [23]. Most likely, YXXL and YXXΦ motifs within the cytoplasmic tails of gE serve as molecular zip codes to direct the protein into transport vesicles in the TGN preordained for transit to their destination in basolateral surfaces [24]. When gE is incorporated in a transport vesicle, the cytoplasmic tail would be oriented toward the cytoplasm (sticking outside of the vesicle membrane), and the gE ectodomain would be oriented toward the lumen (sticking inside of the vesicle). In this arrangement, gE cytoplasmic tail containing the YXXL and YXXΦ motifs may interact with proteins in the cytosol [25]. The YXXΦ/L/V are the best-characterized sorting signals recognized by the adaptor proteins (AP) AP 1, AP-3, and AP-4. These adaptor proteins are localized at the TGN and predicted to generate transport vesicles destined for apical and/or basolateral cell-surfaces [26,27,28]. Therefore, it is possible that the tyrosine-based 563YTVV566 motif in the gE CT of BHV-1 interacted either with AP1 or AP3 adaptor protein and sorted into the transport vesicles destined for the basolateral cell surface.

In the context of a neuron, the apical surface would be the axon-terminal membrane, whereas the basolateral surface would be the cell body and dendritic membranes. Currently, we do not know whether the apical and basolateral sorting of the membrane proteins in MDBK cells correspond, in the context of the highly specialized neurons, to the dendritic (basolateral) and axonal (apical) surfaces, respectively. It remains to be seen whether, like in MDBK cells, BHV-1 gE is expressed in both the axon-terminal and dendritic membranes of infected neurons. If so, then it will be of interest to determine whether or not the gE Y467A, Y563A, and gE Y467A/Y563A localize in the TGN, synaptic vesicles, and dendritic/axon-terminal membranes of infected neurons. Therefore, further investigation concerning these questions is necessary to understand better the role of gE CT and, in particular, the gE CT tyrosine-based motifs in the anterograde axonal neuronal transport.
